# Human Bone Marrow- and Adipose Tissue-derived Mesenchymal Stromal Cells are Immunosuppressive *In vitro* and in a Humanized Allograft Rejection Model

**DOI:** 10.4172/2157-7633.S6-001

**Published:** 2013-11-25

**Authors:** Marieke Roemeling-van Rhijn, Meriem Khairoun, Sander S Korevaar, Ellen Lievers, Danielle G Leuning, Jan NM IJzermans, Michiel GH Betjes, Paul G Genever, Cees van Kooten, Hans JW de Fijter, Ton J Rabelink, Carla C Baan, Willem Weimar, Helene Roelofs, Martin J Hoogduijn, Marlies E Reinders

**Affiliations:** 1 Internal Medicine, Erasmus MC, Rotterdam, The Netherlands; 2 Nephrology, Leiden University Medical Center, The Netherlands; 3 General Surgery, Erasmus MC, Rotterdam, The Netherlands; 4 Immunohematology and blood transfusion, Leiden University Medical Center, The Netherlands; 5 Department of Biology, University of York, York, United Kingdom

**Keywords:** Adipose tissue, Allograft rejection, Bone marrow, Immunomodulation, Mesenchymal stromal cells

## Abstract

**Background:**

Recent studies with bone marrow (BM)-derived Mesenchymal Stromal Cells (MSC) in transplant recipients demonstrate that treatment with MSC is safe and clinically feasible. While BM is currently the preferred source of MSC, adipose tissue is emerging as an alternative. To develop efficient therapies, there is a need for preclinical efficacy studies in transplantation. We used a unique humanized transplantation model to study the *in vivo* immunosuppressive effect of human BM-MSC and adipose tissue-derived MSC (ASC).

**Methods:**

Gene expression of BM-MSC and ASC and their capacity to inhibit activated PBMC proliferation was evaluated. The *in vivo* immunosuppressive effect of BM-MSC and ASC was studied in a humanized mouse model. SCID mice were transplanted with human skin grafts and injected with human allogeneic PBMC with or without administration of BM-MSC or ASC. The effect of MSC on skin graft rejection was studied by immunohistochemistry and PCR.

**Results:**

BM-MSC and ASC expressed TGFβ, CXCL-10 and IDO. IDO expression and acitivity increased significantly in BM-MSC and ASC upon IFN-γ stimulation. IFN-γ stimulated BM-MSC and ASC inhibited the proliferation of activated PBMC in a significant and dose dependent manner. In our humanized mouse model, alloreactivity was marked by pronounced CD45+ T-cell infiltrates consisting of CD4+ and CD8+ T cells and increased IFN-γ expression in the skin grafts which were all significantly inhibited by both BM-MSC and ASC.

**Conclusion:**

BM-MSC and ASC are immunosuppressive *in vitro* and suppress alloreactivity in a preclinical humanized transplantation model.

## Introduction

In the last decades, the interest in Mesenchymal Stromal Cells (MSC) as a cell therapeutic agent has grown substantially [1-5]. These fibroblast-like, plastic adherent cells have multipotent differentiation capacity and while they were originally isolated from bone marrow (BM), they can be isolated from virtually all tissues, including adipose tissue [6-10]. In absence of a specific marker, MSC are commonly defined by a panel of cell surface markers; including CD73, CD90, CD105 and CD166; and their multilineage differentiation capacity [11].

MSC are immunosuppressive cells as indicated by their capacity to inhibit activated T cell proliferation *in vitro* [12,13]. This immunosuppressive potential is enhanced under inflammatory conditions in which IFNγ plays an important role [14,15]. In animal studies, the potential of MSC has been confirmed in multiple disease models for conditions including Graft versus Host Disease (GvHD) [16,17]; ischemia-reperfusion-induced renal injury [18,19]; and liver failure [20,21]. Subsequently, several clinical studies have been conducted evaluating MSC therapy in amongst others GvHD [22]; end stage liver disease [23] and Crohn’s disease [24].

In the field of transplantation, MSC are of great interest due to their potential to inhibit the alloimmune response as well as to contribute to tissue repair. Animal transplant studies showed that MSC are capable to inhibit acute rejection and or to prolong allograft survival [25-28]. As a result, MSC have been applied in kidney transplant recipients [29-31] and a study with MSC in liver transplantation has been initiated [32]. Recent studies, including ours, showed the safety and feasibility of autologous MSC treatment in kidney transplant recipients when using BM-MSC in renal transplant recipients with subclinical rejection or as induction therapy [29-31,33]. While these studies are indicative of an immunomodulatory effect of MSC in human, efficacy of MSC therapy in clinical transplantation remains to be determined.

Currently, BM is the most commonly used source of MSC. However, BM aspiration is an invasive procedure accompanied with donor morbidity. In contrast, adipose tissue can be obtained in a minimal invasive way via lipectomy or liposuction and is therefore emerging as an alternative [34]. Bone marrow has a lower yield of MSC compared to adipose tissue, and adipose tissue derived MSC (ASC) have a higher proliferation capacity than bone marrow derived MSC (BM-MSC) [35-37]. Considering their basic features, BM-MSC and ASC show many resemblances. Both BM-MSC and ASC express the classical MSC markers CD73, CD90, CD105 and CD166 [36,37] although some differences in immunophenotype between BM-MSC and ASC have been reported [34,38,39]. BM-MSC and ASC both display the spindle shape MSC morphology and they are both capable of multilineage differentiation. Immunosuppressive capacities *in vitro* have been acknowledged for BM-MSC [12,40,41] and ASC [6,42-44]. In animals transplant models, both BM-MSC [12,25-27,45,46] and ASC [28] have shown to be beneficial in inhibiting acute rejection and or increasing allograft survival. The efficacy of BM-MSC and ASC in humanized transplant models has however never been investigated. In the present study we focus on the immunomodulatory effects of BM-MSC and ASC *in vitro* and in a humanized skin allograft rejection model.

## Materials and Methods

### Bone marrow and adipose tissue donors

MSC were isolated from either BM or adipose tissue from healthy controls. BM was obtained from 5 hematopoietic stem cell donors after written informed consent as approved by the Medical Ethical Committee of Leiden University Medical Centre as described before [47]. Adipose tissue was obtained from 9 live kidney donors after written informed consent as approved by the Medical Ethical Committee of the Erasmus MC (protocol no. MEC-2006-190).

### BM-MSC isolation and culture

BM was aspirated under general anesthesia. The mononucleated cell (MNC) fraction was isolated by Ficoll density gradient separation and plated in tissue culture flasks at a density of 160 × 10^3^ MNC/cm^2^ in low-glucose Dulbecco’s modified Eagle medium DMEM (Invitrogen, Paisley, UK) supplemented with penicillin/streptomycin (Lonza, Verviers, Belgium) and 10% fetal calf serum (FCS, Thermo Scientific HyClone)). The cultures were maintained at 37°C, 5% CO_2_. The medium was refreshed twice a week. When the MSC cultures became confluent, cells were collected using trypsin (Lonza) and re-plated at a density of 4 × 10^3^ cells/cm^2^ or frozen until further usage.

### ASC isolation and culture

Abdominal subcutaneous adipose tissue was surgically removed under general anaesthesia during donor nephrectomy. Adipose tissue was mechanically disrupted and enzymatically digested with sterile 0.5 mg/ml collagenase type IV (Sigma-Aldrich, St Louis, MO) in RPMI-1640 þ gluta MAX (Invitrogen) and p/s for 30 min at 37.1°C. Cell pellets were resuspended in MSC culture medium, consisting of MEM-α (Sigma-Aldrich(St. Louis, MO, USA) with 1% p/s and 15% fetal bovine serum (BioWhittaker, Verviers, Belgium), transferred to a 175- cm^2^ culture flask (Greiner Bio-one, Essen, Germany) and kept at 37°C and 5% CO_2_. Medium was refreshed twice a week. When ASC cultures became 90% confluent, cells were detached using 0.05% trypsin-EDTA and frozen until further usage.

For experiments, only passage 1-5 BM-MSC and ASC were used. Prior to usage for experiments BM-MSC and ASC were cultured in parallel under the same culture conditions.

### Isolation of peripheral blood mononuclear cells (PBMCs)

PBMCs were collected from buffy coats of healthy blood bank donors (different than the MSC donors). PBMCs were isolated by density gradient centrifugation using Ficoll Isopaque (Pharmacia Amersham, Uppsala, Sweden) and used directly or frozen at −135°C until use.

### Real-time RT-PCR

For evaluation of mRNA gene-expression by BM-MSC and ASC, 100,000 MSC were seeded per well in a 6 well plate in either normal MSC culture medium or MSC culture medium supplemented with 100 ng/ml IFNγ. After 24 hours, MSC cell pellets were harvested.

RNA was isolated and cDNA synthesized as described previously [48]. Quantitative expression was determined by real-time RT-PCR using universal PCR mix (Invitrogen) and Assays- on-demand for IDO (Hs00158627.m1), TGF-β (Hs00171257.m1) and CXCL-10 (Hs 00171042.m1) on a StepOnePlus real time PCR system (Applied Biosystems). Gene-expression was depicted as ratio with GAPDH.

### IDO activity measurements

BM-MSC and ASC were cultured in standard MSC culture medium or culture medium supplemented with 100 ng/ml IFNγ. After 1 week, medium was removed and BM-MSC and ASC were washed twice with 1× PBS. Next, BM-MSC and ASC were cultured for 24 hours in serum free MEM-α (+1% p/s) before medium was harvested. The tryptophan metabolic activity of IDO was determined by measurement of L-kynurenine in the conditioned medium of four unstimulated and four stimulated BM-MSC and ASC cultures. 30% trichloroacetic acid was added to the samples at a 1:3 ratio and after 30 min incubation at 50°C the samples were centrifuged at 12,000 rpm for 5 min. Supernatants were then diluted 1:1 in Ehrlich reagent (200 mg 4-dimethylaminobenzaldehyde (Sigma, St. Louis, MO, USA) in 10 ml of glacial acetic acid) in duplicate in a 96-wells flat bottom plate and absorbance was determined at 490 nm using a multilabel plate reader (VersaMaxTM, Molecular Devices, Sunnyvale, CA, USA). L-kynurenine (Sigma, St. Louis, MO, USA) diluted in unconditioned medium was used as standard.

### Anti-CD3/CD28 lymphocyte stimulation assay

MSC were seeded in round bottom 96-wells plates in MEMα with 10% heat inactivated human serum at 0.125 × 10^4^; 0.25 × 10^4^; 0.5 × 10^4^; 1 × 10^4^ or 2 × 10^4^ cells per well. PBMCs were stimulated with anti-human-CD3 (0.5 ug/5 × 10^5^ cells), anti-human-CD28 (0.5 ug/5 × 10^5^ cells), and goat-anti-mouse antibody (0.5 ug/5 × 10^5^ cells) for cross-linking (all BD Pharmingen).

PBMCs were added to the round-bottom 96-well plates at 5 × 10^4^ cells per well. Proliferation was measured by incorporation of ^3^H-thymidine (0.5 μCi) during 8 h using a beta-plate reader (LKB) on day 3. All experiments were performed in triplicate and medians were used for further analysis.

### Humanized SCID mouse allograft model

#### Animals

CB-17 SCID/mice were obtained from Charles River Laboratories (Wilmington, Massachusetts, USA) and were used at age 8-10 weeks. The Animal Care and Use Committee of the Leiden University Medical Center approved all experiments. Animals were housed under specific pathogen free conditions in individually ventilated cages and allowed free access to food and water throughout the experiments.

#### Humanized SCID model

The humanized skin allograft model was approved by local medical ethical committee and local animal ethical committee. The study is based on the model described by Moulton et al. with slight adaptations [49]. In summary, human abdominal skin was obtained from healthy individuals who underwent elective plastic surgery procedures. Eight-mm punch biopsies were harvested and kept in culture medium and transplanted onto the back of CB-17 SCID mice within 12 hours. The mouse skin was shaved and skin grafts were fixed with a transparent adhesive film (Smith and Nephew, Hoofddorp, Netherlands) and allowed to heal for 4-6 weeks. For humanization, third party peripheral blood mononuclear cells (PBMCs) were isolated from fresh buffy coats, obtained from healthy controls, as described before.

All animals received 100 μl of anti–asialo GM1 by intraperitoneal injection to deplete host NK cells at day-1 (Wako Chemicals USA, Richmond, VA, USA). At day 0, 24 hours after administration of anti-asialo, 3 × 10^8^ PBMCs were transferred by intraperitoneal injection.

MSC were cultured for 1 week in the presence of 100 ng/ml IFNγ (PeproTech EC Ltd, London, UK) before injection into SCID mice.

BM-MSC (0.5 × 10^6^ per infusion, n=8 mice) or ASC (0.5 × 10^6^ per infusion, n=7 mice) were injected under the skin graft at day 0 and 4. Control mice (n=9) received 100 μl saline at the same time points. Animals were sacrificed at day 14 days and skin grafts were harvested. The humanized SCID model is depicted in Figure 3a.

#### Immunohistochemistry

Immunohistochemical staining was performed as described previously [50]. Skin sections (4 μm) of snap-frozen skins grafts were air dried and acetone fixed. Slides were incubated overnight with mouse monoclonal IgG against human leukocytes (CD45; BD Pharminghen, Breda Netherlands) or cytotoxic T-lymphocytes (CD8; Millipore, Amsterdam Netherlands) and rabbit polyclonal anti-CD3 (NeoMarkers, Fremont, California, USA). Antibody binding was detected with horseradish peroxidase (HRP)-labeled goat anti-mouse IgG and goat anti-rabbit IgG (both DAKO, Glostrum, Germany). After washing, sections were incubated with tyramide-fluorescein itransplantationhiocyanate in tyramide buffer (NENTM Life Science Products, Boston, MA, USA), washed and incubated with HRP-labeled rabbit anti-fluorescein itransplantationhiocyanate (DAKO, Glostrum, Germany) and developed with 3,3′-Diaminobenzidine (DAB) (Sigma, St Louis, MO, USA). Sections were counterstained with haematoxylin (Merck, Darmstadt, Germany) and mounted with imsol (Klinipath, Duiven, the Netherlands). Quantification of immunohistochemistry was performed in a blinded manner by assessing 10 consecutive high power fields (magnification, ×200) on each section. Using Image J software, the percentage positive area in each image was determined.

#### Real-time RT-PCR

RNA was isolated from explanted skingrafts using TRIzol Reagent (Life technologies) and RNeasy minikit (Qiagen, Venlo, The Netherlands) according to manufacturer’s recommendations. Quantitative expression was determined by real-time RT-PCR as described above using Assays- on-demand for IFNγ (Hs 00174086.m1), TNF-α (Hs99999043.m1), IL1-β (Hs00174097.m1), IL-6 (Hs 00174131.m1) and GAPDH (Hs 99999905.m1) (all Applied Biosystems, Foster City, CA). Gene-expression was depicted as ratio with GAPDH.

### Statistical analyses

Mann Whitney statistical test was used to test for statistical significance, p value<0.05 was considered statistical significant.

## Results

### Bone marrow and adipose tissue donors

Study tissues were collected from healthy BM donors (n=5, mean age 15 years, range 7-31 years) and healthy adipose tissue donors (n=5, mean age 54 years, range 32-67 years). The expanded MSC from healthy BM and adipose tissue donors all expressed the common MSC markers CD73, CD90, CD105 and CD166 (data not shown).

### BM-MSC and ASC express immunomodulatory genes

**Figure 1:**
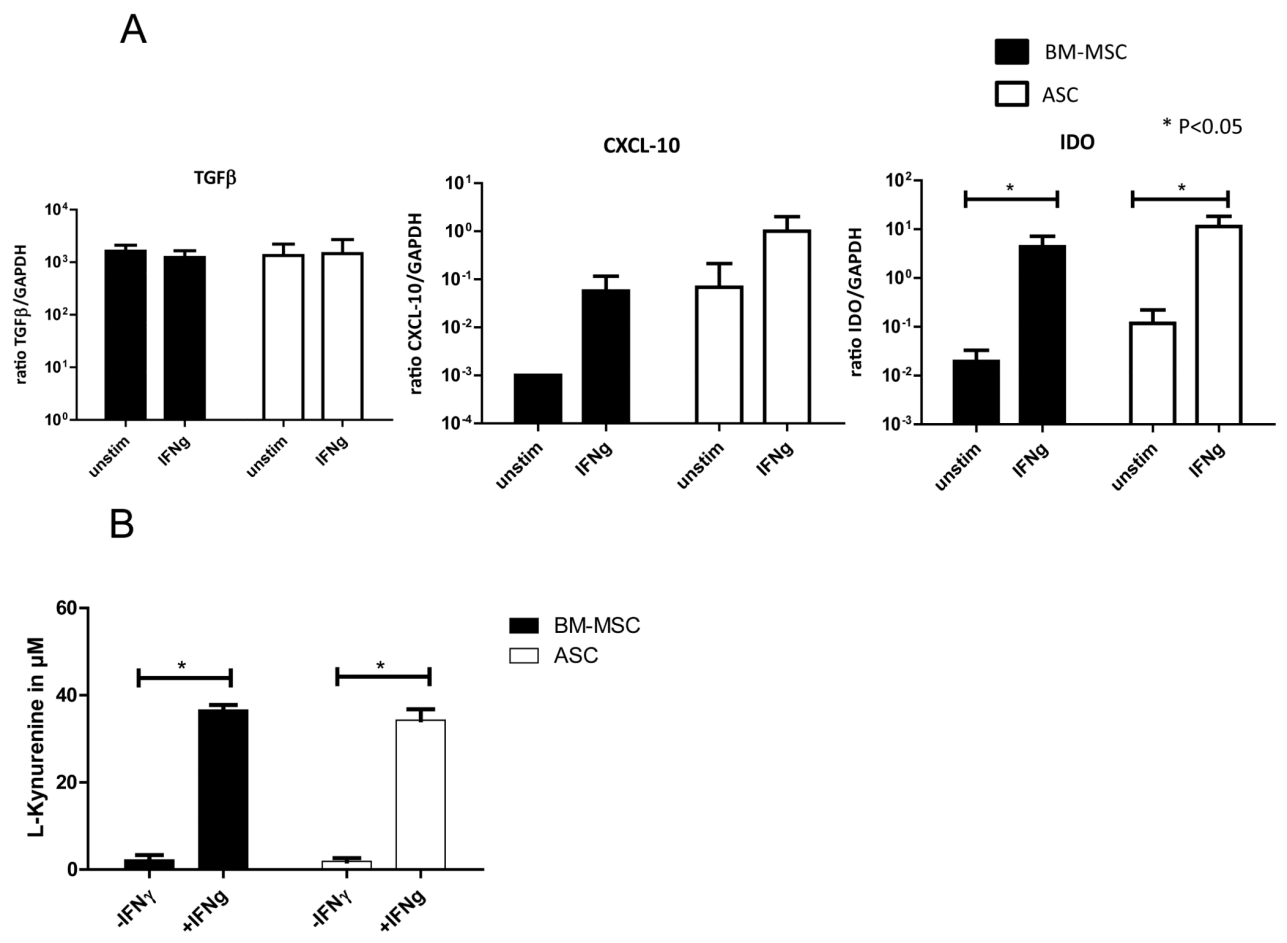
(A) mRNA gene expression of immunomodulatory genes of BM-MSC and ASC with or without IFNγ stimulation, n=5 for BM-MSC and n=5 for ASC. Ratio gene/GAPDH ×1000 is shown. Means (SD) are depicted, *represents P<0.05. (B) Effect of IFNγ stimulation on IDO activity in BM-MSC and ASC determined by measurement of L-kynurenine in medium conditioned with unstimulated or IFNγ stimulated BM-MSC and ASC, n=4 for BM-MSC and n=4 for ASC, means (SD) are depicted, *represents P<0.05.

Both BM-MSC and ASC expressed TGF-β, CXCL-10, and IDO mRNA (Figure 1A). Since MSC are activated under inflammatory conditions, we investigated the effect of the pro-inflammatory cytokine IFNγ on BM-MSC and ASC gene expression. Expression of TGFβ and CXCL-10 mRNA was not affected by 1 week of IFNγ stimulation. However, IDO mRNA expression was increased both in BM-MSC and ASC after IFNγ stimulation.

### IDO activity increases after IFNγ stimulation in BM-MSC and ASC

To evaluate whether increased IDO mRNA expression was translated in enhanced IDO activity upon IFNγ stimulation of BM-MSC and ASC, we studied the concentrations of L-kynurenine in BM-MSC and ASC conditioned medium. In medium conditioned with IFNγ stimulated BM-MSC and ASC, levels of L-kynurenine were significantly higher compared to medium conditioned with unstimulated BM-MSC and ASC (Figure 1B). As L-kynurine is the breakdown product of tryptophan, this indicates an increase of the tryptophan depleting activity of IDO in BM-MSC and ASC after IFNγ stimulation, which is suggestive for immunosuppressive activity of BM-MSC and ASC.

### BM-MSC and ASC have immunosuppressive properties *in vitro*

**Figure 2:**
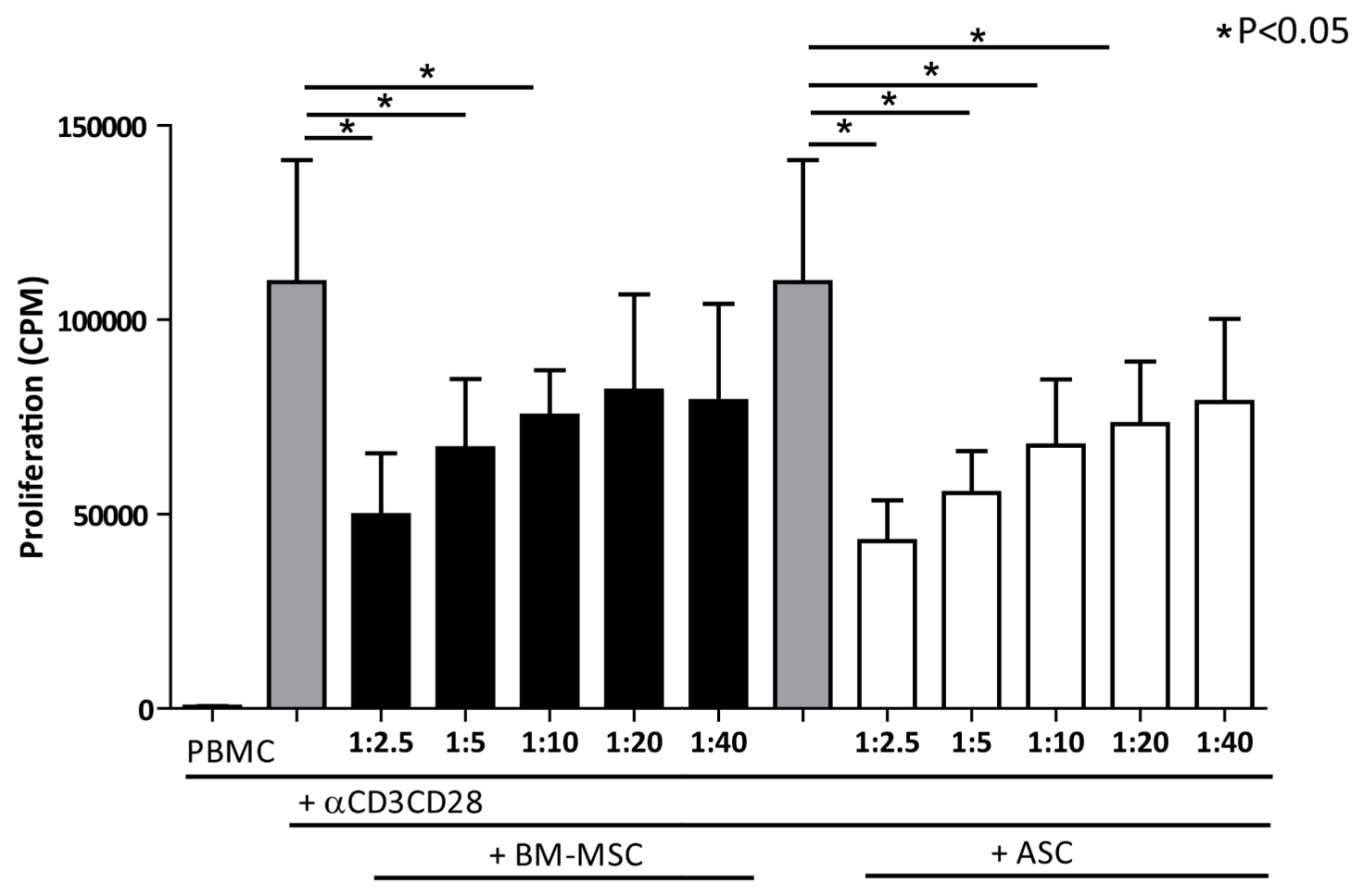
Inhibition of aCD3CD28 stimulated PBMC by INFγ stimulated BM-MSC (n=4) and ASC (n=4). Means (SD) are depicted, *represents P<0.05.

We then compared the immunosuppressive capacity of IFNγ stimulated BM-MSC and ASC. The capacity of these stimulated MSC to inhibit the proliferation of αCD3 CD28 activated PBMCs was evaluated. Both IFNγ stimulated BM-MSC and ASC were capable of significant and dose dependent inhibition of αCD3αCD28 activated PBMC proliferation. The BM-MSC group showed significant inhibition until a MSC: PBMC ratio of 1:10, ASC until a MSC: PBMC ratio of 1:20. No significant difference was detected between BM-MSC and ASC in the percentage inhibition (Figure 2).

### BM-MSC and ASC downregulate alloreactivity *in vivo*

**Figure 3:**
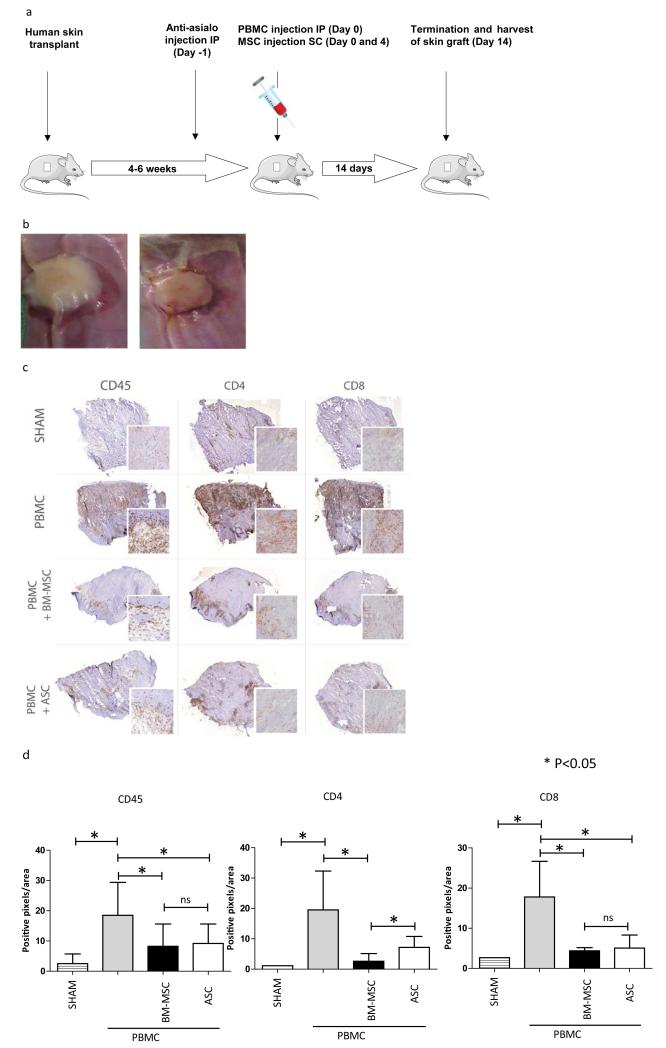

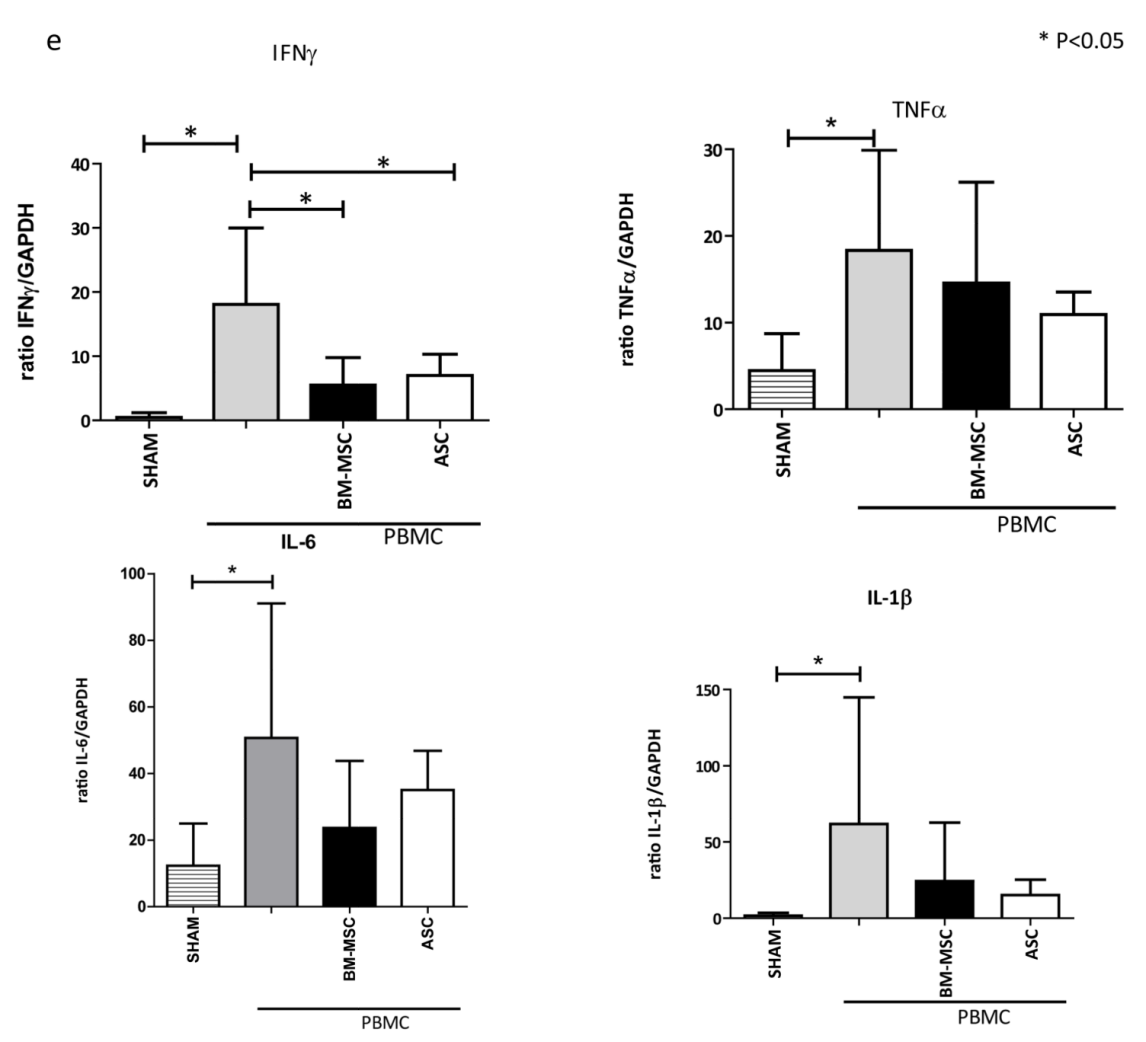
(A) HuSCID mouse model: human skin is transplanted; mouse NK cells are depleted using anti-asialo GM1 injection at day-1; allogeneic PBMC are injected intraperitoneal at day 0; MSC are injected on day 0 and day 4; and after 14 days, mice are sacrificed and skin grafts are harvested. (B) Representative example of engraftment process of skin graft, pictures taken at 12 and 16 days after skin graft transplantation. (C) Immunohistological evaluation of explanted skin grafts. Staining for CD45+, CD4+ and CD8+ T cells of skin grafts explanted from mice receiving only a skin transplant (SHAM group); mice receiving skin graft and PBMC; mice receiving skin graft, PBMC and BM-MSC; and mice receiving skin graft, PBMC and ASC. Representative biopsies are shown. (D) Quantitative evaluation of CD45+, CD4+ and CD8+ cells in biopsies of study groups. Data represent mean (SD), *indicates P<0.05. (E) mRNA gene expression in explanted skin grafts of IFNγ, TNFα, IL-6 and IL-1β of study groups. Ratio gene/GAPDH ×1000 is shown. Data represent mean (SD), *indicates P<0.05.

After confirmation of immunosuppressive capability *in vitro*, we investigated the effect of IFN-γ-stimulated BM-MSC and ASC on leukocyte recruitment into human skin in a huSCID mouse model (Figure 3A). Human skin was transplanted and engrafted well within 6 weeks (Figure 3B). After adoptive transfer of human PBMCs into the mouse by intraperitoneal injection, there was a marked infiltration of CD45+ leukocytes in the skin graft at day 14 (Figures 3C and 3D). Despite this marked leukocyte infiltration in the graft, skin integrity of the huSCID mice remained intact and did not slough. This is probably due to the maintenance of blood flow by the murine vasculature as also described previously [49,50]. The infiltrating CD45+ cells consisted of both CD4+ and CD8+ T cells (Figures 3C and 3D). Mice not receiving an adoptive transfer of human PBMCs did not demonstrate leukocyte infiltration (SHAM group, Figures 3C and 3D). Administration of BM-MSC or ASC at day 0 and day 4 after allogeneic PBMC injection resulted in a significant reduction of CD45+, CD4+ and CD8+ T cell infiltration (Figures 3C and 3D). RT-PCR analysis for expression of various pro-inflammatory human cytokines was performed on the explanted skin grafts of the mice. The mice that had received PBMC revealed a significant increase in mRNA expression of IFNγ, TNFα, IL-6 and IL1-β indicating inflammation compared to mice with skin grafts only (Figure 3E). When mice with engrafted skin grafts were treated with BM-MSC or ASC after PBMC injection, expression of IFNγ significantly decreased (Figure 3E). Expression of TNFα, IL-1β and IL-6 was also reduced after BM-MSC or ASC treatment, although these differences did not reach statistical significance.

## Discussion

We found that MSC derived from bone marrow and adipose tissue was capable of immune suppression *in vitro* and inhibition of T cell mediated alloreactivity *in vivo*. These results are important for future clinical application of MSC. MSC are emerging as a cell therapeutic agent in the field of transplantation with now four completed trails in kidney transplant recipients and multiple trails in preparation [29-31,33,51]. However, currently, there is no consensus considering the optimal source for the isolation of MSC for clinical application.

Our finding that BM-MSC and ASC are both effective indicates that the choice between BM-MSC and ASC for clinical application can be made upon practical considerations. This is important as, despite the numerous studies published on MSC and immune modulation, only a few compared their immunosuppressive capacity. Moreover, in the field of transplantation, no humanized mouse models have been used to evaluate the effect of MSC. In the present study, we first evaluated the *in vitro* immunosuppressive capacities of the MSC. We found that BM-MSC and ASC both express CXCL-10, TGFβ and IDO. The chemoattractive CXCL-10, the immune regulatory TGFβ and antiproliferative IDO are known factors via which MSC excert their immune modulating properties [13,52,53]. Thus, our results confirm the immunosuppressive potential of both types of MSC. Further, as it is known that an inflammatory environment can stimulate the immunosuppressive MSC function [54], we evaluated their response to IFNγ stimulation. Upon IFNγ stimulation, we found that IDO expression and activity was increased in both BM-MSC and ASC. This is in line with the notion that an inflammatory environment, in this study simulated with IFNγ, can potentiate MSC efficacy in both BM-MSC and ASC.

In αCD3αCD28-induced PBMC proliferation assays we found BM-MSC and ASC both capable of dose dependent inhibition. These results confirm previous data showing that BM-MSC and ASC have the capacity to inhibit proliferation of alloantigen and mitogen activated PBMC [17,39,55] and justifies examination of these cell populations in a preclinical transplant model.

Multiple animal studies have shown the potential of BM-MSC [12,25-27,45,46] and ASC [28] to ameliorate ischemia reperfusion injury or alloreactivity. Yet, as mentioned before, human BM-MSC and human ASC have not been evaluated in a humanized transplant model.

Issa et al. used such a humanized model for evaluation of regulatory T cell (Tregs) therapy. This model enabled the authors to study the effect of ex vivo-expanded Tregs and found them capable of inhibition of skin graft rejection [56]. Here, for the first time, we used such a unique humanized mouse model for evaluation of MSC treatment in allograft rejection. We found that BM-MSC and ASC were effective in inhibiting alloreactivity. Alloreactivity was marked by recruitment of CD45+ cells which consisted of CD4+ and CD8+ T cells and IFNγ, TNFα, IL-1β and Il-6 mRNA expression was increased in the explanted skin grafts. Local administration of BM-MSC as well as ASC reduced inflammation by inhibiting the recruitment of T cells and decreasing IFNγ, TNFα, IL-6 and IL-1β expression in the skin grafts. These results support the use of BM-MSC and ASC to treat transplant rejection.

In conclusion, the immunosuppressive potential of BM-MSC and ASC has been highlighted in the present study. Our experiments confirm the immunosuppressive capacities of BM-MSC and ASC *in vitro*. By extending our investigations to a humanised transplant model our data underscore the potential of BM-MSC and ASC therapy in clinical transplantation.

## Abbreviations

ASC: Adipose Tissue Derived Mesenchymal Stromal Cells
BM: Bone Marrow
BM-MSC: Bone Marrow Derived Mesenchymal Stromal Cells
MSC: Mesenchymal Stromal Cells
PBMC: Peripheral Blood Mononuclear Cells
